# The Successful Treatment of Pneumonia with Oxygen

**Published:** 1893-09

**Authors:** 


THE SUCCESSFUL TREATMENT OF PNEUMONIA WITH OXYGEN (BRIN).—Robert W. Bateman (*The Lencet;* June 8, 1892).

Dr. Bateman reports the case of a 63-year-old patient, in whom an attack of influenza was followed by a lobar pneumonia, involving the lower lobe of the left lung. In a few days, consolidation of the right apex developed. From this time on inhalations of oxygen gas were given, lasting ten minutes at a time, and repeated during the course of the day. This procedure was followed by a decided diminution of the cyanosis and amelioration of all symptoms; a decided stimulating effect was also noticeable after fourteen days' time. The inhalations were prolonged for twenty minutes.

Incidentally, the author calls attention to the fact that, after an attack of influenza, inflammatory processes take on a decided asthenic condition, while decidedly stimulating drugs, such as ether, ammonia, senega, and hyoscyamus, are called for when a distressing cough exists. At the same time, the application of cataplasms to the entire throax is essential; this same should encircle the chest.

According to the writer, the irregular pneumonia following influenza should be treated differently; he also asserts that the influenza poison penetrating into the lung can produce diseased processes in different portions of the lung.

It seems to be preferable (according to Brin's system) to direct the current of oxygen into the mouth after closing the valve, and not to fill the bag which is connected with the cylinder. After removal of the poultice, it is well to apply tincture of iodine to the chest—*American Medico-Surgical Bulletin.*

